# Supplementary material for: Divergent roles of FT-like 9 in flowering transition under different day lengths in Brachypodium distachyon
Source: Nat Commun. 2019 Feb 18;10:812. doi: 10.1038/s41467-019-08785-y (PMC6379408; doi:10.1038/s41467-019-08785-y)
Supplement: Supplementary file 1 — Supplementary Information [file 41467_2019_8785_MOESM1_ESM.pdf]

Supplementary Information

Divergent roles of FT-like 9 in flowering transition under different day lengths in *Brachypodium distachyon*

Qin *et al.*

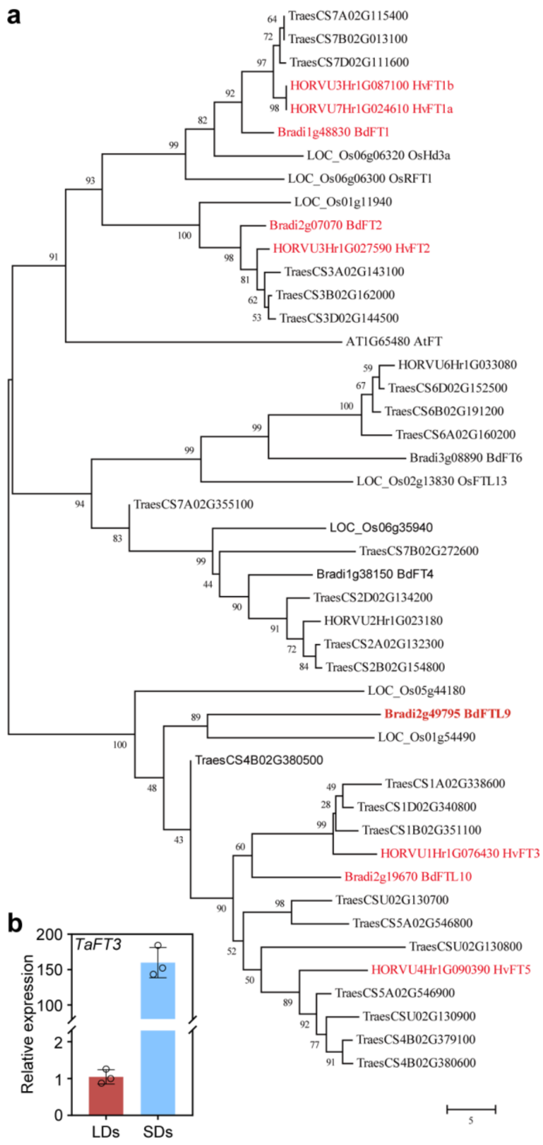

Supplementary Figure1 FTL9 is induced under SDs in *Brachypodium distachyon*

(a) A phylogenetic tree of FT-like proteins in temperate grasses and other plants. Alignments sequences were produced by CLUSTALW, and used for phylogenetic analysis. The midpoint-rooted phylogenetic tree was constructed by MEGA program using the Neighbor-Joining method. Bootstrap numbers shown at nodes are percentage of 1000 replicates. The gene numbers for barley and wheat were from Ensembl Plants database, with the reference genome as Hv_IBSC_PGSB_v2 and IWGSC RefSeq v1.0, respectively. FTL9 in *B. distachyon* is highlighted by bold characters.

(b) qRT-PCR analysis of *FT3* orthologous gene expressions in wheat under SDs and LDs.

UBC18 was used as an internal control for normalization of qRT-PCR results. Error bars indicate standard deviation of three biological replicates.

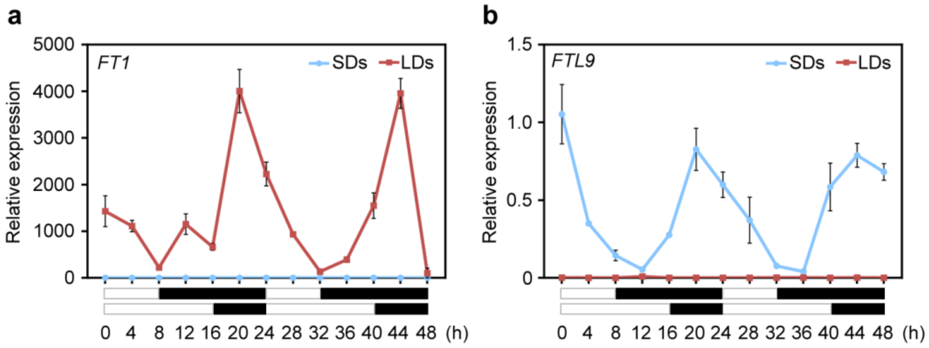

Supplementary Figure 2 FT1 and FTL9 is controlled by day length with a oscillatory rhythm in temperate grasses

(a) Diurnal expression patterns of *FT1* in *B. distachyon* under SDs (8h of light/16h of dark) and LDs (16h of light/8 h of dark). Each point means the average of three technical replicates, and the error bars represent repeat SD. The white and black bars along the horizontal axes indicate light and dark periods, respectively. The numbers below the horizontal axes are the time points.

(b) Diurnal expression patterns of *FTL9* in *B. distachyon* under under SDs (8h of light/16h of dark) and LDs (16h of light/8 h of dark). Each point means the average of three technical replicates, and the error bars represent repeat SD. The white and black bars along the horizontal axes indicate light and dark periods, respectively. The numbers below the horizontal axes are the time points.

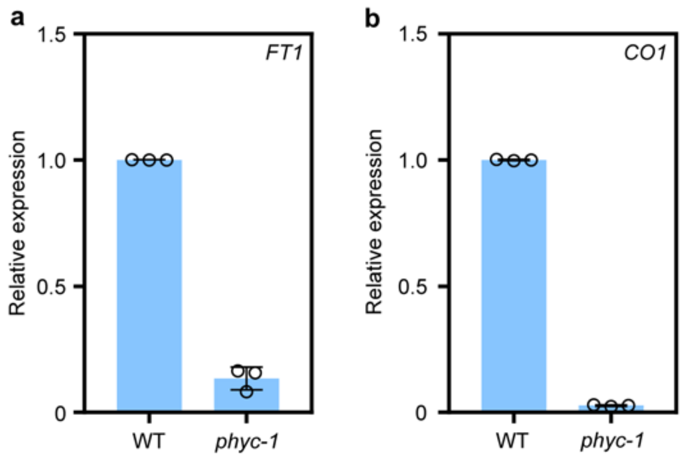

Supplementary Figure 3 *FT1* and *CO1* expressions are decreased in *phyc* mutants

qRT-PCR analysis of *FT1*(a) and *CO1*(b) in wild-type Bd21-3 and *phyc-1* homozygous mutants under LDs. *UBC18* was used as an internal control for normalization of qRT-PCR results. Error bars indicate standard deviation of three biological replicates.

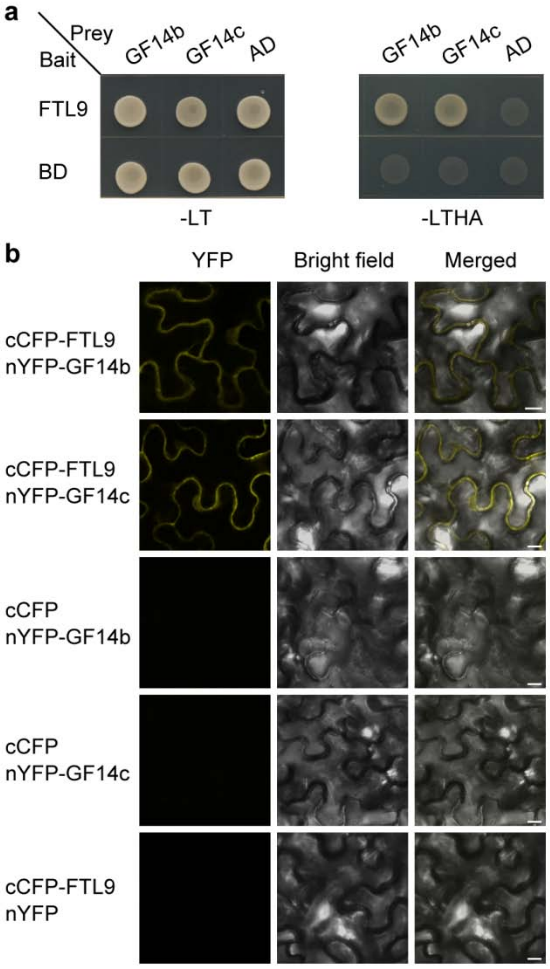

Supplementary Figure 4 FTL9 interacts with 14-3-3 family proteins

(a) Interaction analysis of FTL9 with 14-3-3 family proteins GF14b and GF14c in yeast cells. Yeast growth on selective media without Leu, Trp, His and Ade (-LTHA) represents positive interactions.

(b) Interactive determination of FTL9 with 14-3-3 family proteins GF14b and GF14c by BiFC assays. Scale bar, 10μm.

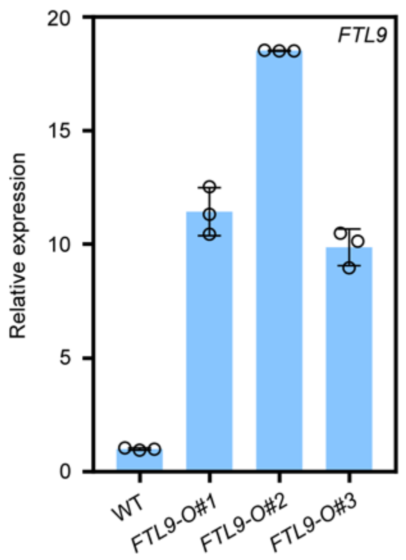

Supplementary Figure 5 Generation of *FTL9* over-expression plants in *B. distachyon*

qRT-PCR analysis of *FTL9* in wild-type Bd21 and *FTL9* over-expressing lines under LDs. *UBC18* was used as an internal control for normalization of qRT-PCR results. Error bars indicate standard deviation of three biological replicates.

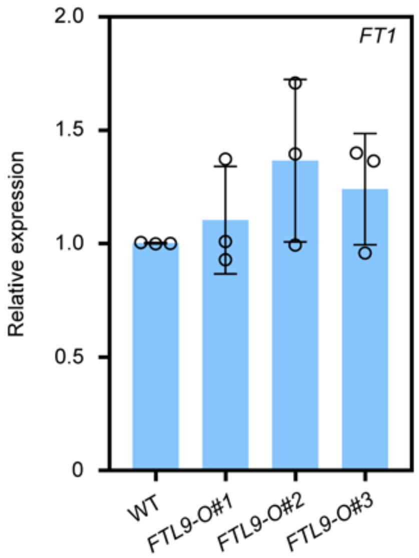

Supplementary Figure 6 *FT1* is not significantly increased in *FTL9* over-expression plants under SDs

qRT-PCR analysis of *FT1* in wild-type Bd21 and *FTL9* over-expressing lines under SDs. *UBC18* was used as an internal control for normalization of qRT-PCR results. Error bars indicate standard deviation of three biological replicates.

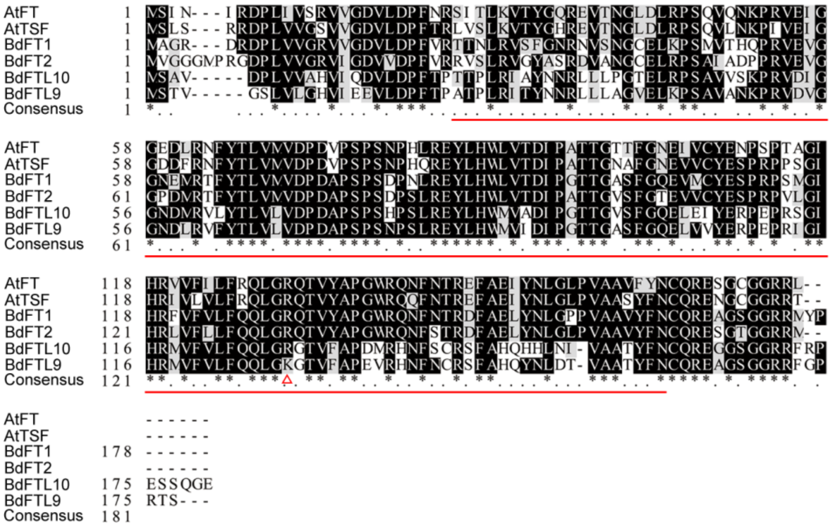

Supplementary Figure 7 Protein sequence alignment of FT proteins in *B. distachyon* and *A.thaliana*

PEBP domain of indicated FTs are marked by the red line. Lysine (Lys, K) at residue 128 of FTL9 is marked by a red triangle. Amino acids underlined by stars means the same amino acids and underlined by dots means those with similar property.

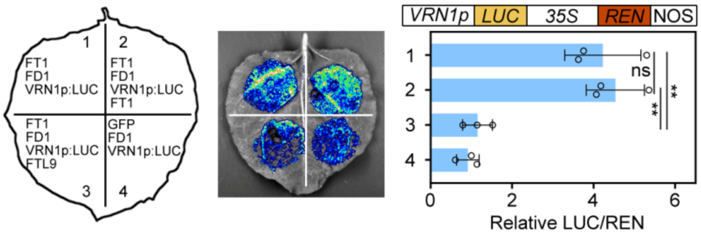

Supplementary Figure 8 Additional FT1 cannot attenuate the binding ability of FT1-FAC to *VRN1 cis*-elements

Left: A schematic diagram of constructs of effectors and reporters used in the assay. Middle: Different combinations of effectors and the reporter transiently introduced in *N. benthamiana* leaves. Right: Representative photograph of firefly luciferase fluorescence signals when the indicated reporter and effectors were introduced in *N. benthamiana* leaves.

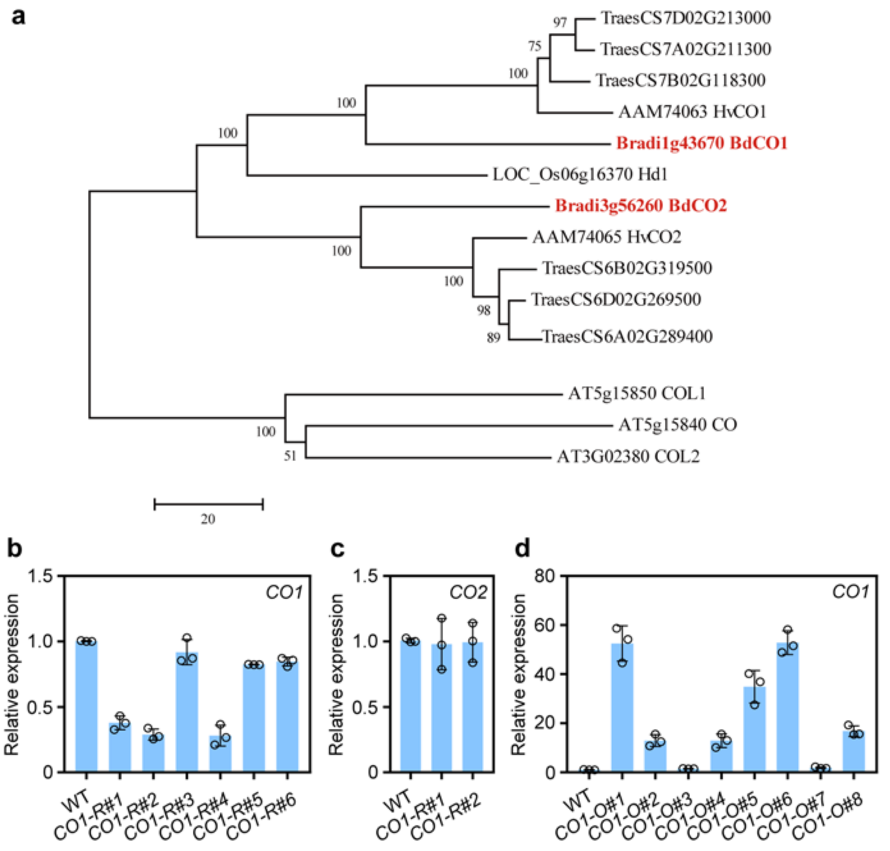

Supplementary Figure 9 Generation of *CO1* over-expression and knocked-down transgenic plants

(a) A phylogenetic tree of CO-like proteins in temperate grasses and other plants. Alignments sequences were produced by CLUSTALW, and used for phylogenetic analysis. The midpoint-rooted phylogenetic tree was constructed by MEGA program using the Neighbor-Joining method. Bootstrap numbers shown at nodes are percentage of 1000 replicates. The gene numbers for barley and wheat were from Ensembl Plants database, with the reference genome as Hv_IBSC_PGSB_v2 and IWGSC RefSeq v1.0, respectively.

(b) qRT-PCR analysis of *CO1* in wild-type and *CO1* knocked-down lines under LDs. *UBC18* was used as an internal control for normalization of qRT-PCR results. Error bars indicate standard deviation of three biological replicates.

(c) qRT-PCR analysis of *CO2* in wild-type and two *CO1* knocked-down lines under LDs. *UBC18* was used as an internal control for normalization of qRT-PCR results. Error bars indicate standard deviation of three biological replicates.

(d) qRT-PCR analysis of *CO1* in wild-type and *CO1* over-expressing lines under LDs. *UBC18* was used as an internal control for normalization of qRT-PCR results. Error bars indicate standard deviation of three biological replicates.

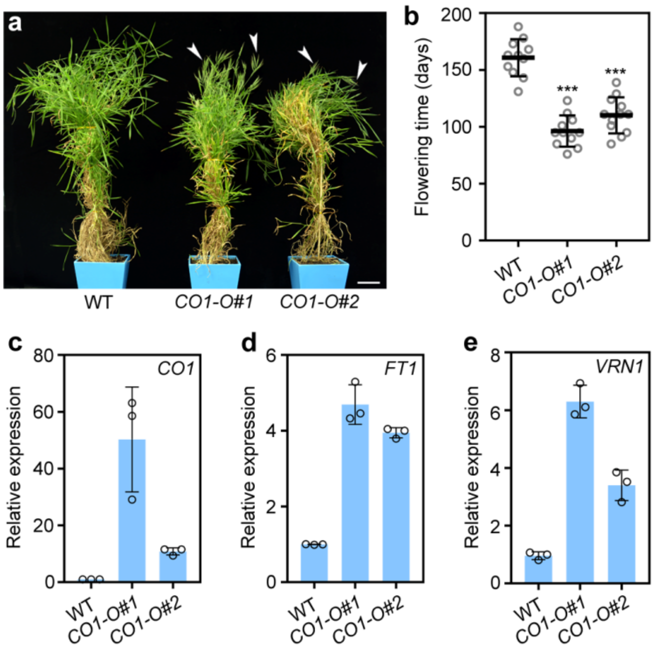

Supplementary Figure 10 Over-expression of *CO1* triggers early flowering by activation of *FT1* in *B. distachyon*.

(a) Typical flowering phenotypes of wild-type Bd21-3 and *CO1* over-expressing plants under SDs. White arrows point to spikes. Scale bar, 5cm.

(b) Flowering time of wild-type Bd21-3 and *CO1* over-expressing plants under SDs. At least 10 plants for each line were scored. (Student's *t* test, ***P <0.001).

(c) qRT-PCR analysis of *CO1* in wild-type Bd21-3 and *CO1* over-expressing lines under SDs. *UBC18* was used as an internal control for normalization of qRT-PCR results. Error bars indicate standard deviation of three biological replicates.

(d) *FT1* expression examination in wild-type Bd21-3 and *CO1* over-expressing plants under SDs. *UBC18* was used as an internal control for normalization of qRT-PCR results. Error bars indicate standard deviation of three biological replicates.

(e) *VRN1* expression determination in wild-type Bd21-3 and *CO1* over-expressing plants under SDs. *UBC18* was used as an internal control for normalization of qRT-PCR results. Error bars indicate standard deviation of three biological replicates.

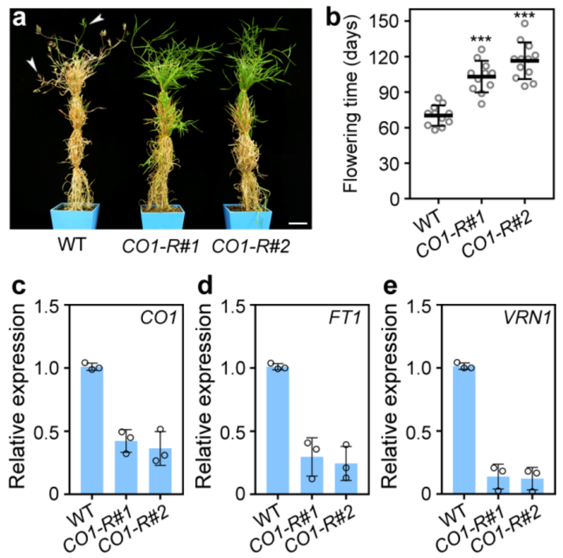

Supplementary Figure 11 *FT1* is decreased in *CO1-RNAi* transgenic plants.

(a) Typical flowering phenotypes of wild-type Bd21-3 and *CO1-RNAi* plants under LDs. White arrows point to spikes. Scale bar, 5cm.

(b) Flowering time of wild-type Bd21-3 and *CO1-RNAi* plants under LDs. At least 10 plants for each line were scored. (Student's *t* test, ***P <0.001).

(c) qRT-PCR analysis of *CO1* in wild-type Bd21-3 and *CO1-RNAi* lines under LDs. *UBC18* was used as an internal control for normalization of qRT-PCR results. Error bars indicate standard deviation of three biological replicates.

(d) *FT1* expression examination in wild-type Bd21-3 and *CO1-RNAi* plants under LDs. *UBC18* was used as an internal control for normalization of qRT-PCR results. Error bars indicate standard deviation of three biological replicates.

(e) *VRN1* expression determination in wild-type Bd21-3 and *CO1-RNAi* plants under LDs. *UBC18* was used as an internal control for normalization of qRT-PCR results. Error bars indicate standard deviation of three biological replicates.

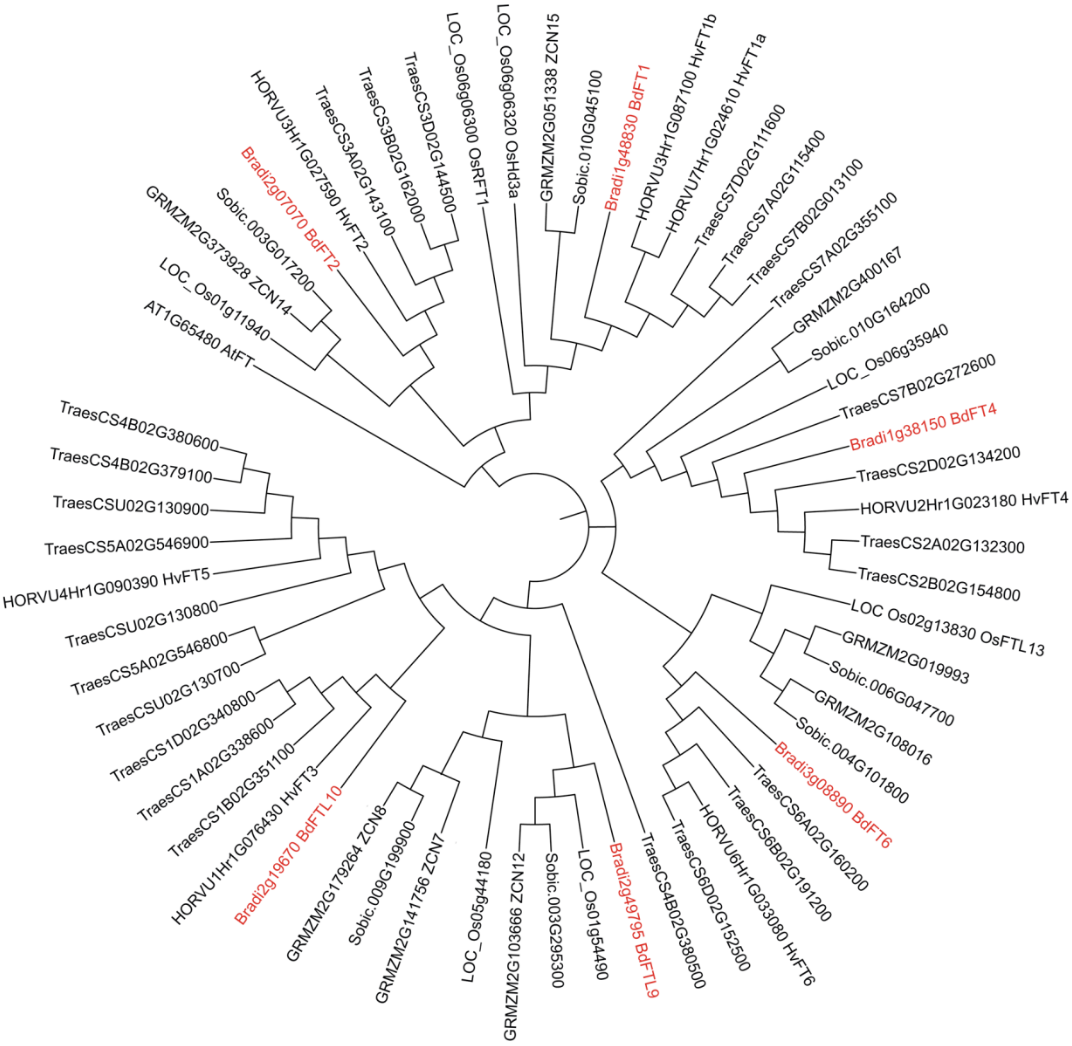

Supplementary Figure 12 A phylogenetic tree of FT-like proteins in temperate grasses, rice, sorghum and maize

Alignments sequences were produced by CLUSTALW, and used for phylogenetic analysis. The midpoint-rooted phylogenetic tree was constructed by MEGA program using the Neighbor-Joining method. Bootstrap numbers shown at nodes are percentage of 1000 replicates. The gene numbers for barley and wheat were from Ensembl Plants database, with the reference genome as Hv_IBSC_PGSB_v2 and IWGSC RefSeq v1.0, respectively.

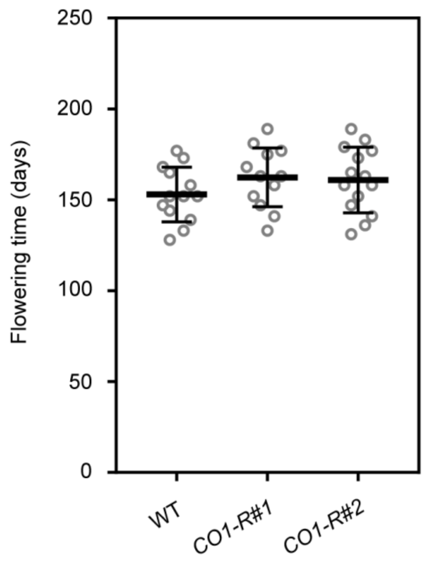

Supplementary Figure 13 Down-regulation of *CO1* cannot trigger a premature flowering under SDs in *B. distachyon*.

Flowering time of wild-type Bd21-3 and *CO1-RNAi* plants under SDs. At least 10 plants for each line were scored.

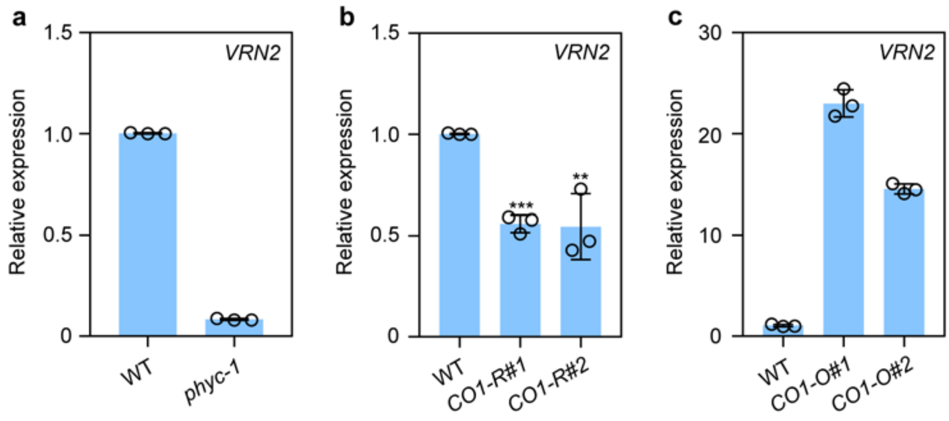

Supplementary Figure 14 VRN2 is a candidate regulator of *FTL9* in *B. distachyon*.

(a) qRT-PCR analysis of *VRN2* in wild-type Bd21-3 and *phyc-1* mutants under LDs. *UBC18* was used as an internal control for normalization of qRT-PCR results. Error bars indicate standard deviation of three biological replicates.

(b) qRT-PCR analysis of *VRN2* in wild-type Bd21-3 and *CO1 RNAi* lines under LDs. *UBC18* was used as an internal control for normalization of qRT-PCR results. Error bars indicate standard deviation of three biological replicates.

(c) qRT-PCR analysis of *VRN2* in wild-type Bd21-3 and *CO1* over-expression lines under LDs. *UBC18* was used as an internal control for normalization of qRT-PCR results. Error bars indicate standard deviation of three biological replicates.

Supplementary Table 1.Primers used in this study

Primer Name	Primer Sequence(5′ to 3′)
**Gene cloning and vector construction**	
FTL9-1390-F	TTACTTCTGCACTAGGTACCATGTCCACTGTGGGATCAT
FTL9-1390-R	GAATTCCCGGGGATCCTTAAGAAGTTCGGGGCCCGAAT
FTL9/Cas9-gRT	TGGCAAACAAGCCAAGAGTTGATGTGTTTTAGAGCTAGAAAT
FTL9/Cas9-U3T1	AAAACACATCAACTCTTGGCTTGTTTGCCACGGATCATCTGC
CO1-OE-F	CAAAAAAGCAGGCTTCATGAATTATAATTTCAACAGCTCCG
CO1-OE -R	CAAGAAAGCTGGGTCGAACCATGGAACGGTGCTGTA
CO1-RNAi-F	CAAAAAAGCAGGCTTCCTGTTATGCTGACTGCGCG
CO1-RNAi-R	CAAGAAAGCTGGGTCTGCTGCCTCGGCAAGTAAAG
FTL9-BD-F	CATGGAGGCCGAATTCATGTCCACTGTGGGATCAT
FTL9-BD-R	GCAGGTCGACGGATCCTTAAGAAGTTCGGGGCCCGAAT
FD1-AD-F	GGAGGCCAGTGAATTCATGGCGGCAATGGAAGACGATG
FD1-AD-R	CGAGCTCGATGGATCCGAATGGCGCCGAGAGCACCCT
GF14b-AD-F	GGAGGCCAGTGAATTCATGTCGGCACCTGCGGAGCTT
GF14b-AD-R	CGAGCTCGATGGATCCCTGCCCATCACCAGAGTCACC
GF14c-AD-F	GGAGGCCAGTGAATTCATGTCGCGGGAGGACAATGTT
GF14c-AD-R	CGAGCTCGATGGATCCCTGGCCCTCGCCAGCTTCACCTT
FTL9-BiFC-F	CAAAAAAGCAGGCTTCATGTCCACTGTGGGATCAT
FTL9-BiFC-R	CAAGAAAGCTGGGTCAGAAGTTCGGGGCCCGAATCTT
FD1-BiFC-F	CAAAAAAGCAGGCTTCATGGCGGCAATGGAAGACGATG
FD1-BiFC-R	CAAGAAAGCTGGGTCGAATGGCGCCGAGAGCACCCT
GF14b-BiFC-F	CAAAAAAGCAGGCTTCATGTCGGCACCTGCGGAGCTT
GF14b-BiFC-R	CAAGAAAGCTGGGTCCTGCCCATCACCAGAGTCACC
GF14c-BiFC-F	CAAAAAAGCAGGCTTCATGTCGCGGGAGGACAATGTT
GF14c-BiFC -R	CAAGAAAGCTGGGTCCTGGCCCTCGCCAGCTTCACCTT
FTL9-cLUC-F	ACGCGTCCCGGGGCGGTACCATGTCCACTGTGGGATCAT
FTL9-cLUC-R	AGCTCTGCAGGTCGACTTAAGAAGTTCGGGGCCCGAATCT
FD1-nLUC-F	CGGGGGACGAGCTCGGTACCATGGCGGCAATGGAAGACGATG
FD1-nLUC-R	ACGAGATCTGGTCGACGAATGGCGCCGAGAGCACCCT
pGreen-VRN1p-F	CGGTATCGATAAGCTTCGAGCAACCTCCTCATATGTAGCG
pGreen-VRN1p-R	TAGAACTAGTGGATCCCTCCTCCCGGAATCGAGACACGAA
pGreen-FTL9p-F	CGGTATCGATAAGCTTGTCAACCGCGCCGCATAAATCAC
pGreen-FTL9p-R	TAGAACTAGTGGATCCGTTTTCTGTGAGAAAAAGTATAACC
FT1-35S-F	CAAAAAAGCAGGCTTCATGGCCGGGAGGGACAGGGAC

FT1-35S-R	CAAGAAAGCTGGGTCGGGGTACATCCTCCTGCCGCC
FTL9-35S-F	CAAAAAAGCAGGCTTCATGTCCACTGTGGGATCAT
FTL9-35S-R	CAAGAAAGCTGGGTCAGAAGTTCGGGGCCCGAATCTT
FD1-35S-F	TAAGCCCGGGGGATCCATGGCGGCAATGGAAGACGATG
FD1-35S-R	GCAGGTCGACTCTAGATCAGAATGGCGCCGAGAGCACCCT
FTL9-F1	ATGTCCACTGTGGGATCA
FTL9-FT1PEBP-R1	TCACCCTCAGGTTGGTTGTTGGTGTAAATGGATCCAAAAC
FTL9-FT1PEBP-F2	TTGGATCCATTTACACCAACAACCAACCTGAGGGTGAGCT
FTL9-FT1PEBP-R2	CCAGCTTCCCTTTGACAGTTGAAGTAGACGGCGGCGACGG
FTL9-FT1PEBP-F3	CGCCGTCTACTTCAACTGTCAAAGGGAAGCTGGTTCTG
FTL9-R3	AGAAGTTCGGGGCCCGAATCTT
FTL9-KpnI-F	ctaggtaccATGTCCACTGTGGGATCAT
FTL9-XbaI-R	gcatctagaAGAAGTTCGGGGCCCGAATCTT
FTL9-K128R-R1	TGCAAAAACTGTCCCccgGCCAAGTTGTTGGAAAAGCACA
FTL9-K128R-F2	GCcggGGGACAGTTTTTGCACCAGAAGTGCGACACAACTT
FT1-F1	ATGGCCGGGAGGGACAGGGAC
FT1-FTL9PEBP-R1	ATTCTGAGGGGGGTAGCCCGGACGAACGGGTCCAGCACGT
FT1-FTL9PEBP-F2	TGGACCCGTTCGTCCGGGCTACCCCCCTCAGAATAACCT
FT1-FTL9PEBP-R2	CCGGCCTCGCGCTGGCAGTTAAAATATGTGGCAGCCACAG
FT1-FTL9PEBP-F3	CTGCCACATATTTTAACTGCCAGCGCGAGGCCGGATCCGG
FT1-R3	GGGGTACATCCTCCTGCCG
FT1-KpnI-F	ctaggtaccATGGCCGGGAGGGACAGGGAC
FT1-XbaI-R	gcatctagaGGGGTACATCCTCCTGCCG
**qRT-PCR**	
FT1-F	CATCACAATCCATGGGCCCATACAG
FT1-R	GCAGTCGCCGTCATTTACATGTGTG
FT2-F	TGGTTGTGATGGTCCGTTTG
FT2-R	AGACAGAACCGACTTGCTAGAAATTAC
FTL10-F	ACTAAGGGAGTACTTGCACTGGATG
FTL10-R	CTAAACCTTCTTCCACCCGATCC
FT4-F	CGAGTGGCCTATACCCTAGTTATGA
FT4-R	TTGGGCTCTCGTATGTTACGATCTC
FTL9-F	GGTTCTAGGCCATGTCATAGAG
FTL9-R	ATCCACCAGTACCAGCGTGTAGAAC
FT6-F	CTCAGCGTAACATATACACTAGTCA
FT6-R	CCGTACGTGTCATCTGTTGATGCT

VRN1-F	GCTCTGCAGAAGGAACTTGTGG
VRN1-R	CGCTGCTGGGCGATTACTGAT
FUL2-F	GCTACTCGTATGCTGAAAAGGC
FUL2-R	CAGTGACCTCTCCTTCTTTTGT
CO1-F	TGATAGCCACATCTACAACAACC
CO1-R	TGGAATCTGTGTAAGCACTGAC
CO2-F	TCCGTATTATGACAACGCCAC
CO2-R	GGTCTTCTGAAACTTTCTTGCCT
VRN2-F	ATGCATGAGAGAGAGGCGAAGG
VRN2-R	TCGTAGCGGATCTGCTTCTCGTAG
UBC18-F	GGAGGCACCTCAGGTCATTT
UBC18-R	ATAGCGGTCATTGTCTTGCG
TaFT3-F	TCACTAAGGGAGTACTTGCACTGGA
TaFT3-R	TGCCTAGTTGCTGGAACAGCAC
TaActin-F	ACCTTCAGTTGCCCAGCAAT
TaActin-R	CAGAGTCGAGCACAATACCAGTTG
